# Pathology and pathogenesis of cutaneous lesions in beef cattle associated with buffalo fly infestation

**DOI:** 10.3389/fvets.2022.971813

**Published:** 2023-01-18

**Authors:** Muhammad Noman Naseem, Rachel Allavena, Ali Raza, Constantin Constantinoiu, Michael McGowan, Conny Turni, Muhammad Kamran, Ala E. Tabor, Peter James

**Affiliations:** ^1^The University of Queensland, Queensland Alliance for Agriculture and Food Innovation, Centre for Animal Science, St. Lucia, QLD, Australia; ^2^School of Veterinary Science, The University of Queensland, Gatton, QLD, Australia; ^3^College of Public Health, Medical and Veterinary Sciences, James Cook University, Townsville, QLD, Australia; ^4^School of Chemistry and Molecular Biosciences, The University of Queensland, St. Lucia, QLD, Australia

**Keywords:** haematobia, *Stephanofilaria*, buffalo fly, skin lesions, histopathology

## Abstract

*Haematobia irritans exigua*, commonly known as buffalo fly, is the major hematophagous ectoparasite of north Australian cattle herds. Lesions associated with buffalo fly infestation are generally alopecic, hyperkeratotic, or scab encrusted wounds with variable hemorrhagic ulceration. Buffalo flies can transmit a filarial nematode, *Stephanofilaria* sp., which has been implicated in the pathogenesis of buffalo fly lesions, but *Stephanofilaria* infection has not been detected in all lesions suggesting that other causal factors may be involved. This study characterized the pathology of buffalo fly lesions to identify the role of *Stephanofilaria* in lesion development, as well as to identify other potential agents. Lesion biopsies were collected from north and south Queensland and tested for the presence of *Stephanofilaria* by qPCR. Each lesion was scored grossly (0–4) for hemorrhage, ulceration, exudation, and alopecia. Lesions were also scored microscopically (0–4) for epidermal and dermal damage and inflammatory characters. *Stephanofilaria* infection was detected in 31% of lesion biopsies. Grossly, *Stephanofilaria*-infected lesions had significantly larger lesion area and higher scores for alopecia and hyperkeratosis than lesions where no nematodes were found (*P* < 0.05). Histologically, epidermal, dermal, and adnexal damage was significantly higher in *Stephanofilaria* infected lesions than lesions without nematodes. Eosinophils, macrophages, and lymphocytes were significantly more abundant in *Stephanofilaria* positive lesions as compared to negative lesions. This study also noted bacterial infection with colonies of coccoid bacteria, observed in skin sections from 19 lesions. Grossly, lesions with bacterial infection had significantly higher ulceration scores compared to *Stephanofilaria* positive lesions, and histologically epidermal disruption was significantly greater in bacteria-infected lesions. We found no evidence of bacteria or *Stephanofilaria* infection in 49% of the lesions assessed and tissue damage patterns and eosinophilic inflammation suggested hypersensitivity to buffalo fly feeding as a possible cause of these lesions. These findings suggest that although the presence of *Stephanofilaria* infection may increase the severity of lesion pathology, it is not essential for lesion development. These outcomes also suggest a potential role of bacteria and hypersensitivity in pathogenesis of some lesion. A better understanding of buffalo fly lesion etiology will contribute to the optimal treatment and control programmes.

## 1. Introduction

Flies from the genus *Haematobia* are obligate hematophagous ectoparasites that feed mainly on cattle and buffalo (1). Two major and closely related species of this genus are the buffalo fly (*Hematobia irritans exigua*) (BF) prevalent in the tropical and subtropical parts of Asia, Australia and other parts of Oceania, and the horn fly (*Haematobia irritans irritans*) (HF) widespread throughout Europe, Africa and the Americas (1). Buffalo flies (BFs) are considered a major pest affecting animal production and welfare, particularly in northern Australian herds (2). Infestation with BFs is frequently accompanied by the development of skin lesions associated with BF feeding that occur mainly near the medial canthus of the eye, along the lateral and ventral neck and on the abdomen. These lesions can range from raised dry, alopecic, hyperkeratotic or scab encrusted to severe haemorrhagic ulcerated areas (3, 4) and occur in up to 95% of northern Australian herds (5). When surveyed, northern dairy farmers noted BF as a problem on 91% of farms and animal welfare aspects due to BF infestation were noted as the most serious BF-related issue (6). Often the key concern of cattle producers are the lesions associated with BF feeding because of their visual appearance (most particularly the open and suppurating wounds) and the associated irritation to the animal (3). Buffalo fly lesions can also penetrate the dermis, affecting hide quality, cattle saleability, and can increase animal susceptibility to secondary infections (7).

In Australia, BFs transmit an unnamed species of filarial nematode, *Stephanofilaria* sp., which has been associated with the pathogenesis of BF lesions (5, 7, 8). This nematode is closely related to, or potentially the same species as *Stephanofilaria stilesi* vectored by horn flies (HFs)in the northern hemisphere and South America (3, 9). However, *Stephanofilaria* nematodes and their microfilariae were detected in only 40% of the lesions examined histologically in a study by Johnson et al. (5) and Naseem et al. (10) found that only 11% of 120 BF lesions assessed were positive for *Stephanofilaria* infection by qPCR. In addition, nematode distribution was limited to northern and central Queensland, and qPCR testing found no *Stephanofilaria* in either lesions or BF from southern Queensland although BF-associated lesions are prevalent in these areas (10).

Although lesion development is associated with BF feeding, there is a poor correlation between BF numbers and lesion development (7, 11) suggesting that there are additional contributing factors. In addition, although HFs have been reported to vector *Stephanofilaria stilesi* causing granular abdominal dermatitis, udder and teat lesions in cattle in North and South America (9, 12–14), the nematode was not found in all cases (14, 15). Thus these lesions were suggested to be potentially due to hypersensitivity induced by HF feeding (16) and infection with *Staphylococcus* spp. bacteria vectored by HFs (17). These observations taken together suggest that *Stephanofilaria* infection may not be essential for the pathogenesis of BF lesions, and that other causal factors may be involved.

In this study, we utilized a recently developed *Stephanofilaria* specific qPCR (18) to identify the presence of nematodes in lesions, and characterized and compared the gross and microscopic pathology of BF-associated lesions with various pathogens present to clarify the key factors involved in the pathogenesis of these lesions.

## 2. Material and methods

### 2.1. Skin lesion sample collection and *Stephanofilaria* testing

Lesion samples (*n* = 86) were collected from skins of recently slaughtered cattle at a commercial abattoir in north Queensland (*n* = 62) as well as from biopsies from live cattle (*n* = 24). All abattoir samples were from lesions near the medial canthus of the eyes whereas biopsies from live cattle were from two herds kept in southern Queensland (Pinjarra Hills −27.50^O^S, 152.91^O^E and Forest Hill −27.60^O^S, 152.39 ^O^E) and were collected from the neck (*n* = 17), shoulder (*n* = 2), belly (*n* = 3) and from near the eye (*n* = 2). All biopsies were taken from the center of the lesion using 8 mm sterile skin punches (Paramount Surgimed Ltd., New Delhi, India) together with a control skin biopsy from unaffected skin 3–4 cm away from each lesion. A sub sample was taken from each of the 86 samples collected and confirmed as positive or negative for *Stephanofilaria* by TaqMan^TM^ qPCR assay (18). All samples were preserved in 10% neutral buffered formalin until histological processing. These studies were conducted under The University of Queensland Animal Ethics approval no. 2021/AE000054.

### 2.2. Gross examination and scoring

The initial gross appearance of each lesion was scored at the time of sample collection according to a *de novo* scoring scheme shown in Table 1. Each lesion was scored (0–4) grossly for the presence of ulceration, exudation, hemorrhage, alopecia, scab formation and hyperkeratosis. All lesions were also photographed alongside a measurement scale before collection of the biopsies and the area of each sampled lesion was measured from photographs using an online tool SketchAndCalc (https://www.sketchandcalc.com/).

**Table 1:** Grading scales for scoring gross changes in buffalo fly lesions.

**Parameter**	**Observation**	**Score**
Hemorrhage	Absent	0
	< 10% of lesion area	1
	11–40% of lesion area	2
	41–80% of lesion area	3
	>80% of lesion area	4
Ulceration	Absent	0
	< 10% of lesion area	1
	11–40% of lesion area	2
	41–80% of lesion area	3
	>80% of lesion area	4
Exudation	Absent	0
	Sero-haemorrhagic	1
	Fibrous	2
	Fibro-purulent	3
	Purulent	4
Scab	Absent	0
	< 10% of lesion area	1
	11–40% of lesion area	2
	41–80% of lesion area	3
	>80% of lesion area	4
Hyperkeratosis	Absent	0
	Periphery (poorly circumscribed outline)	1
	Periphery (well define outline)	2
	Thick on the periphery and protruding to center	3
	Cover more than 80% of the lesion	4
Alopecia	Absent	0
	< 10% of lesion area	1
	11–40% of lesion area	2
	41–80% of lesion area	3
	>80% of lesion area	4

The total gross lesion score was calculated as the sum of each parameter score.

### 2.3. Histological processing

Skin biopsies were dehydrated and cleared using an automated Tissue Processor (Shandon Excelsior ES, Thermofisher Scientific, Waltham, MA, USA). The biopsies were then embedded in paraffin using a tissue embedding machine (Leica EG1160, Leica Biosystem, Wetzlar, Germany). A 4 μm thick section was taken from each biopsy using a manual microtome (Leica RM2235, Leica Biosystem, Wetzlar, Germany) and stained with hematoxylin and eosin. Staining was performed on an auto-stainer (Leica ST5020, Leica Biosystems, Wetzlar, Germany) according to the manufacturer's instructions. Each section was scanned (Leica Aperio CS2, Leica Biosystems, Wetzlar, Germany) and examined on a computer screen.

### 2.4. Histological examination and scoring

All 86 samples were reviewed for the presence of *Stephanofilaria* adult nematodes or microfilariae, and bacteria. For quantitation of the tissue damage, all epidermal and dermal changes were scored by the scoring systems given in Table 2. Epidermal changes including epidermal disruption, crust over epidermis, hyperkeratosis, acanthosis and spongiosis were scored individually for each sample and a total epidermal damage score for each sample was calculated by summing the individual score of all parameters. Similarly dermal damage was scored for adnexal destruction, endothelial activity, vascular changes and collagenolysis, and the total dermal damage was determined for each section by summing the individual scores of all assessed dermal parameters. Differential inflammatory cell scores and total inflammatory response in each lesion were scored according to the scoring scheme shown in Table 3. The scoring schemes for histological changes in this study were created with reference to observations of the biopsies from unaffected skin areas processed in this study. All parameters for histological changes were scored in ten different fields selected randomly from three fields each from the left and right side, and four from the center of each biopsy. A final score was calculated as an average of the ten individual field scores.

**Table 2:** Grading scales for scoring histological changes in buffalo fly lesions.

**Parameter**	**Magnification/field area**	**Observation**	**Score**
**Epidermal changes**			
Epidermal disruption	100X/1.9 × 1.030 mm	Absent	0
		< 10% of lesion area cover	1
		11–40% of lesion area cover	2
		41–80% of lesion area cover	3
		>80% of lesion area cover	4
Spongiosis	200X/950 × 515 μm	Absent	0
		Focal (1-10)/field	1
		Multifocal (>10)/ field	2
		Locally extensive	3
		Diffused throughout the biopsy	4
Acanthosis	100X/1.9 × 1.030 mm	Normal epidermis (40-60 μm thick or 4-5 cell layers)	0
		Twice the thickness of the normal epidermis	1
		Three times the thickness of the normal epidermis	2
		Four times the thickness of the normal epidermis	3
		More than 5 times the thickness of the normal epidermis	4
Crust over epidermis	100X/1.9 × 1.030 mm	Absent	0
		Serous	1
		Serocellular crust (fine thin band)	2
		Serocellular crust (thick band)	3
		Haemorrhagic crust	4
Hyperkeratosis	100X/1.9 × 1.030 mm	Normal stratum corneum (4–8 μm thick)	0
		Twice the thickness of the normal st. corneum	1
		Three times thickness to the normal st. corneum	2
		Four times thickness to of the normal st. corneum	3
		More than 5 times the thickness of the normal st. corneum	4
**Dermal changes**			
Adnexal destruction	100X/1.9 × 1.030 mm	Normal (HF^a^, 5-6; SG^b^, 1-2; Sb-G^c^, 4-5)	0
		(HF, 3-4; SG, 1-2; Sb-G, 2-3)/field	1
		(HF, 2-3; SG, 1; Sb-G, 1-2)/field	2
		(HF, 1-2; SG, 1; Sb-G, 1)/ field	3
		No adnexal structure observed	4
Number of vessels	200X/950 × 515 μm	Not observed	0
		1–2/ fields	1
		3–4/ fields	2
		Greater than 4/in at least 5 fields	3
		Present throughout section	4
Hemorrhage	200X/950 × 515 μm	Absent	0
		1–3 haemorrhagic foci	1
		Over 4 haemorrhagic foci	2
		Multifocal (≥10) hemorrhage	3
		Diffuse hemorrhages	4
Collagenolysis	100X/1.9 × 1.030 mm	Absent	0
		< 10% of the specimen	1
		11–40% of the specimen	2
		41–80% of the specimen	3
		> 80% of the specimen	4

a Hair follicle,

b Sweat gland,

c Sebaceous gland. The total epidermal and dermal damage score was calculated as the sum of each parameter score for epidermis and dermis damage respectively.

**Table 3:** Grading scales for scoring inflammatory cells in buffalo fly lesions.

**Cell type/Response**	**Score**				

	**0**	**1**	**2**	**3**	**4**
Neutrophils	1–2/hpf^*^	3–7/hpf	8–15/hpf	>15/hpf	Heavy Infiltrate
Lymphocytes					
Plasma cells					
Eosinophils					
Macrophages					

* hf = High powered (400X) field. A total inflamatory score was calculated as the sum of each inflammatory cell type score.

### 2.5. Statistical analysis

All the gross and histological scores were compared between different groups using the Mann–Whitney *U*-test (19) and the lesion areas were compared by two tailed *t*-tests in GraphPad Prism version 9.1.0 (GraphPad Software, La Jolla, CA; by www.graphpad.com). The level of statistical significance was *P* < 0.05.

## 3. Results

### 3.1. Detection of potential causal factors

**Figure 1:**
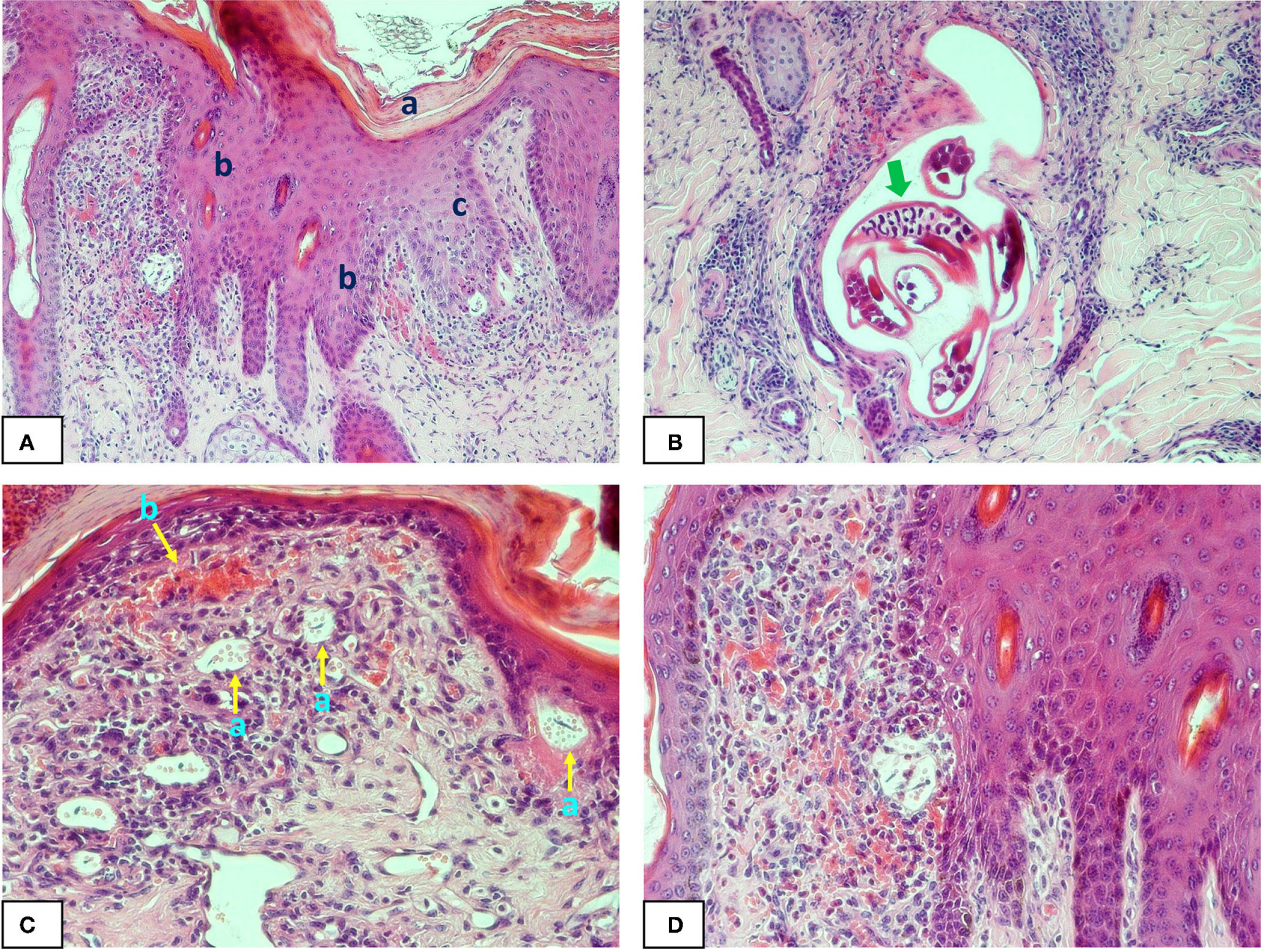
Photomicrographs of buffalo fly lesion histology: **(A)** shows hyperkeratosis (a) acanthosis (b) and spongiosis (c) in the epidermal layer of a *Stephanofilaria* infected lesion (100X); **(B)** shows adult *Stephanofilaria* in a cyst-like structure surrounded by inflammatory cells. The arrow shows the microfilariae within the gravid uterus of an adult female nematode (200X); **(C)** Shows microfilariae close to the epidermis and enclosed in a round to oval-shaped vitelline membrane that also contains numerous small spherical eosinophilic bodies (a) and hyperaemia (b) with extensive inflammation in the superficial dermis (200X); **(D)** shows acanthosis, severe hyperaemia and eosinophilic inflammation in *Stephanofilaria*-infected lesion (200X).

Of 62 lesion samples collected from north Queensland, 27 were positive for *Stephanofilaria* infection by TaqMan^TM^ qPCR while the adult nematodes or its microfilariae or both were detected in the histological sections of only 14 samples. Of these 14 histologically positive samples, adult nematodes and microfilariae were observed in seven and four respectively, while both were observed in three samples (Figure 1). The number of adult *Stephanofilaria* nematodes ranged from 1 to 11 in individual sections, and microfilariae ranged from 5 to 15. No false PCR positive samples were recorded and none of the 24 lesions tested from south Queensland was positive for *Stephanofilaria* infection by either qPCR or histological examination.

**Figure 2:**
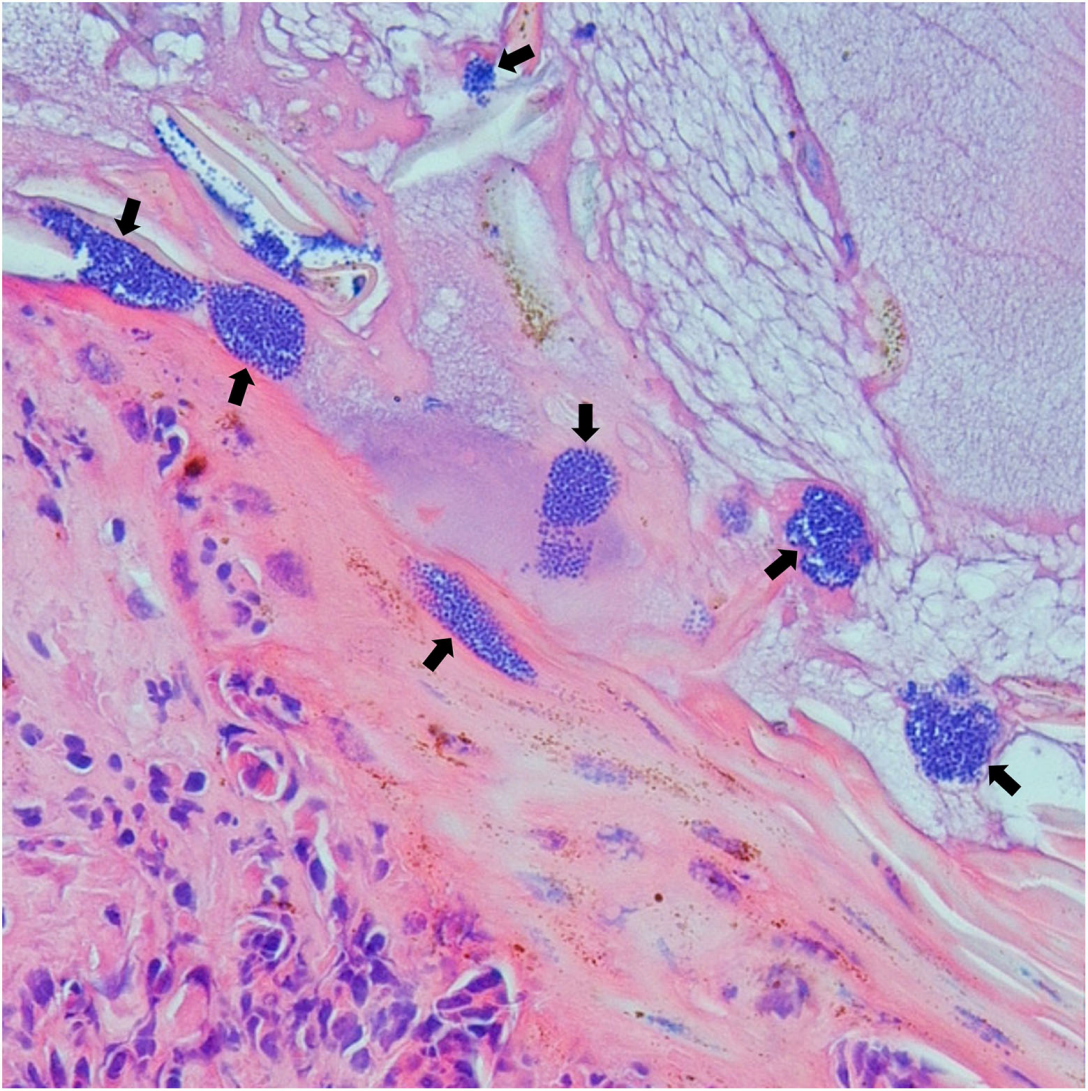
Photomicrograph (400X) of buffalo fly lesions shows multiple clusters of coccoid shaped bacteria (arrow) within the superficial crust.

Multiple clusters of ~1.5 μm purple-stained cocci were observed within the superficial serocellular crust of 19 lesion samples (Figure 2). This included 11 samples from south Queensland and eight from north Queensland. Of these bacteria-positive samples, only two were also positive for *Stephanofilaria* by qPCR.

### 3.2. Gross pathology of BF lesions

**Figure 3:**
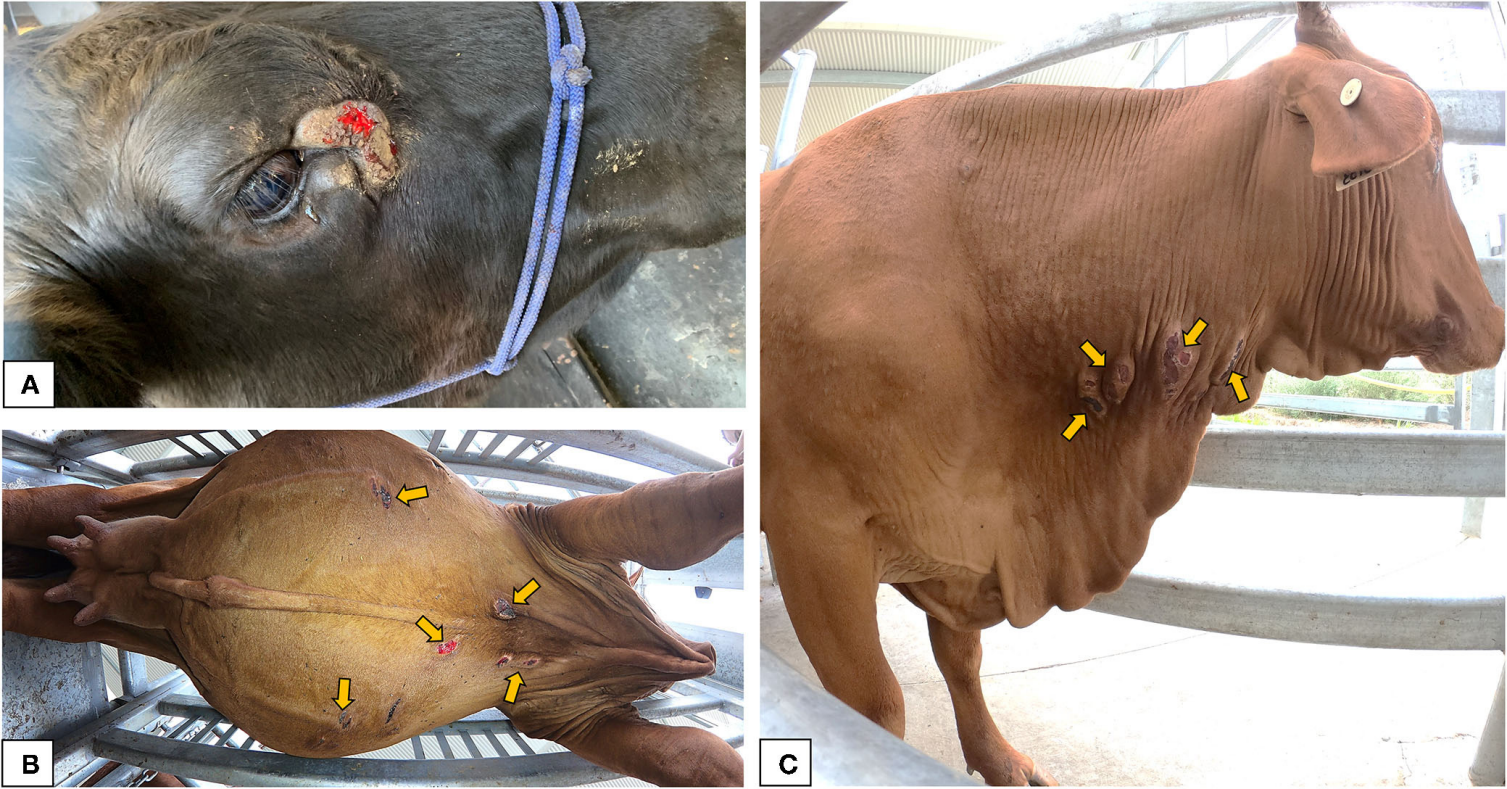
Gross appearance of the buffalo fly lesions: **(A)** lesion adjacent to the medial canthus of the eye in a Brangus steer indicating raised, circumscribed, hairless, ulcerative area partially covered with scab forming from the periphery; **(B)** multiple ulcerative to scab encrusted lesions near the ventral midline of a Droughtmaster cow; **(C)** multiple raised scabbed lesions on the neck of a Droughtmaster cow.

The BF lesions examined ranged from dry, hyperkeratotic hairless areas to severe open suppurative wounds with hemorrhagic or scab encrusted surface (Figure 3). Body lesions including lesions sampled from the neck, dewlap and belly, had areas ranging from 5.14 to 40 cm^2^, while the lesions sampled near the eyes of cattle had lesion areas ranging from 3.04 to 54.44 cm^2^. There were no obvious differences in the gross pathology of lesions sampled from different anatomical locations.

**Figure 4:**
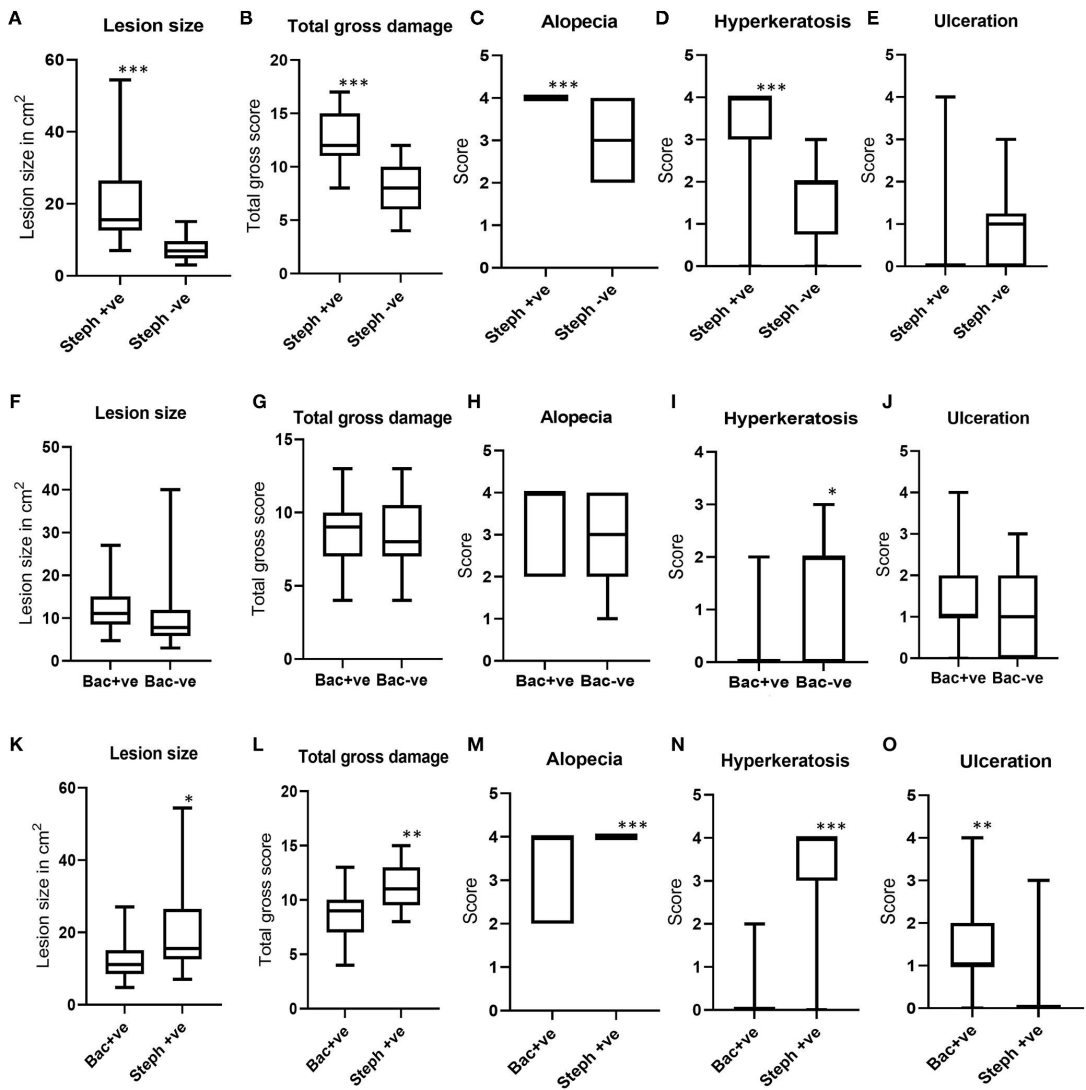
Boxplots showing the distribution of lesion areas and pathology scores for total gross damage, alopecia, ulceration, and hyperkeratosis. **(A–E)** Show gross pathology comparisons between *Stephanofilaria* positive (Steph +ve) and negative (Steph -ve) lesions; **(F–J)** Show gross pathology comparisons between bacteria positive (Bac +ve) and negative (Bac-ve) lesions; **(K–O)** Show gross pathology comparisons between Steph +ve and Bac +ve lesions (*P < 0.05, **P < 0.01, ***P < 0.001).

When the lesion areas and scores for total gross damage, alopecia, ulceration and hyperkeratosis from north Queensland were compared between BF lesions which tested positive (*n* = 25) and negative (*n* = 29) for *Stephanofilaria* infection with PCR, *Stephanofilaria* positive lesions had significantly larger lesion areas (ranging from 7.02 to 54.44 cm^2^) as compared to negative lesions (3.04 to 15.07 cm^2^) (Figure 4A). This comparison excluded two lesions that were positive for both *Stephanofilaria* and bacterial infection to avoid any confounding effects due to bacterial infection. Total gross damage, alopecia and hyperkeratosis were significantly higher in *Stephanofilaria* positive lesions (Figures 4B–D). Notably, all of the animals in the *Stephanofilaria* positive group had a score of 4 for alopecia (>80% of the lesion area affected), whereas the occurrence of alopecia was more variable (score 2–4) in the *Stephanofilaria* negative group. There was only one animal with ulceration score >0 in the *Stephanofilaria* positive group and this animal had severe (score 4) ulceration whereas in the *Stephanofilaria* negative group, more animals had a score of 1–3 for ulceration, but none had a score 4 (Figure 4E).

In the comparison between BF lesions with bacterial infection (*n* = 17) and without bacterial infection (*n* = 42), a non-significant difference was noted in lesion areas between lesions positive (ranged 4.77–27 cm^2^) and negative (3.04 to 40 cm^2^) for bacterial infection (Figure 4F). The scores for total gross damage, alopecia and ulceration were not significantly different between these two groups (Figures 4G, H, J). Lesions with no bacterial growth had significantly higher hyperkeratosis as compared to bacteria-infected lesions (Figure 4I).

When the gross pathology scores for BF lesions positive for *Stephanofilaria* but not bacteria (*n* = 25) were compared with lesions observed with bacterial infection but not *Stephanofilaria* (*n* = 17), *Stephanofilaria*-infected lesions had significantly larger lesion areas (7.02 to 54.44 cm^2^) as compared to bacterial infected lesions (4.77 to 27 cm^2^) (Figure 4K). The scores for total gross damage, alopecia and hyperkeratosis were also significantly higher in *Stephanofilaria*-infected lesions (Figures 4L–N), whereas bacteria-infected lesions had significantly higher ulceration scores (Figure 4O). For this comparison, there was only one animal in the *Stephanofilaria* group with ulceration (score 3) whereas in the bacteria affected lesions ulceration was more common with most animals affected (only one animal with score 0) and scores ranging from 0 to 4.

### 3.3. Microscopic pathology of BF lesions

The most consistent differences in the epidermis between lesion-affected and unaffected skin included the degree of hyperkeratosis, acanthosis, spongiosis, epidermal disruption and formation of a serocellular crust of varying thickness. There were also varying degrees of cellular infiltration in lesion-affected areas with cell composition including necrotic epidermal cells, neutrophils, and eosinophils (Figure 1A). Epidermal disruption was most commonly observed in sections biopsied from body lesions from south Queensland. Changes observed in the dermis included varying degrees of adnexal destruction, endothelial reactivity, vascular changes and infiltration of inflammatory cells. Acute lesions had moderate to severe superficial dermal collagenolysis whereas varying degrees of fibrosis were observed in chronic wounds indicating the commencement of scarring. Cellular infiltrate was predominately eosinophils along with macrophages, neutrophils and lymphocytes.

Adult *Stephanofilaria* nematodes were observed up to 0.5–2.5 mm deep in the dermis, mostly within cysts formed at the base of the damaged hair follicles (Figure 1B). In two lesion sections, adult nematodes were observed close to the epidermis with necrotic tracts in the dermal layer suggesting nematode migration through the dermis. Microfilariae were mostly observed close to the epidermis in the dermal papillae and rete pegs and enclosed in a round to oval-shaped vitelline membrane that also contained numerous small spherical eosinophilic bodies (Figures 1C, D). Similar membrane-enclosed microfilariae were also observed in the uteri of gravid female nematodes. Eosinophils were dispersed throughout the superficial dermal layer with markedly abundant numbers near *Stephanofilaria* adults and microfilariae.

**Figure 5:**
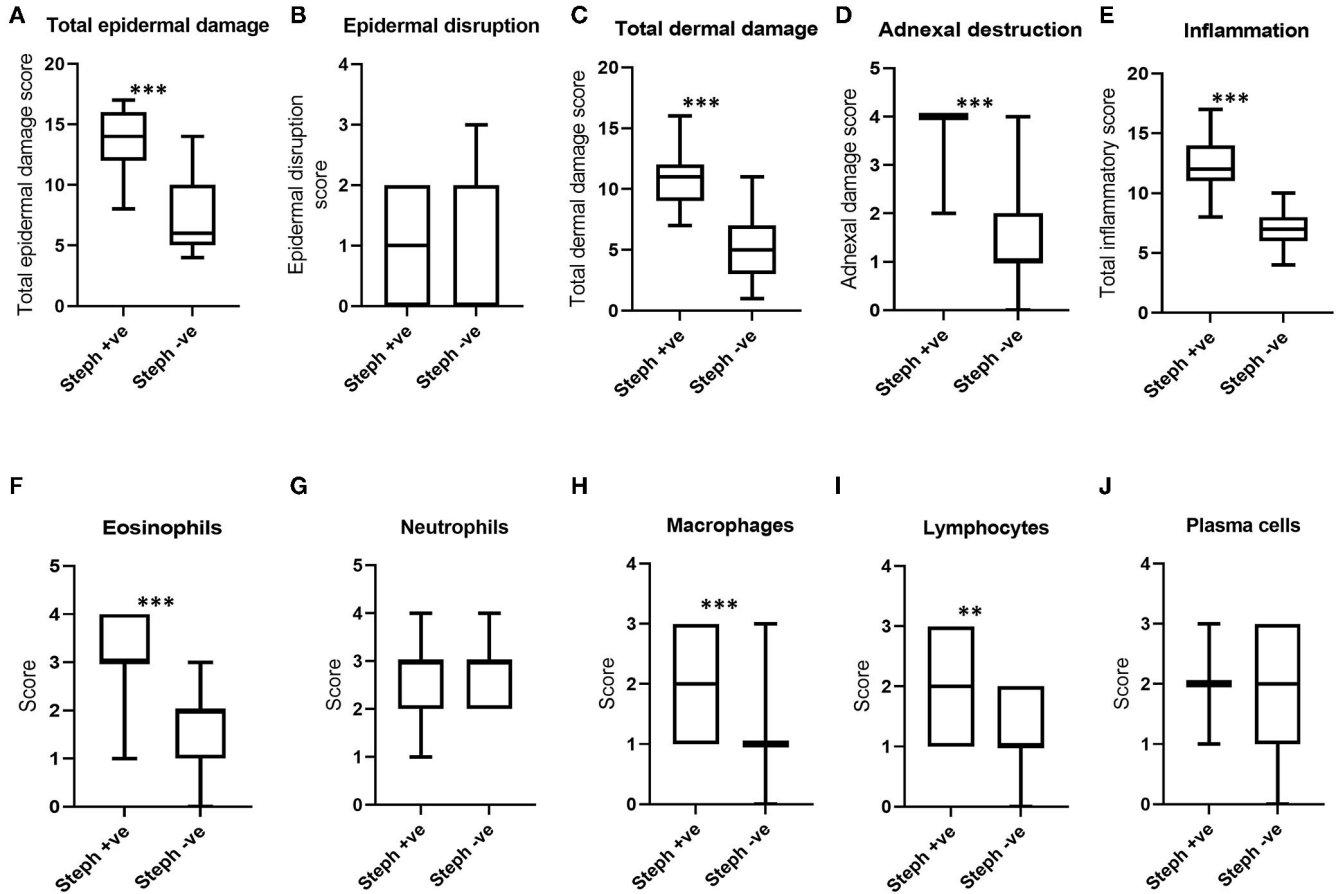
Boxplots **(A–D)** show the distribution of scores for total epidermal damage, epidermal disruption, total dermal damage and adnexal destruction scores for *Stephanofilaria* positive (Steph +ve) and negative (Steph -ve) lesions. The boxplots **(E–J)** show the total inflammation and individual inflammatory cell scores of these two groups (^**^P < 0.01, ^***^P < 0.001).

To characterize the microscopic pathology of BF lesions with different causal factors, histopathological scores were compared among lesions grouped according to potential causal factors.When histopathological scores for north Queensland lesions qPCR positive for *Stephanofilaria* were compared with lesions without *Stephanofilaria* infection, total epidermal damage in *Stephanofilaria* positive lesions was significantly higher than in the negative lesions (Figure 5A). Epidermal disruption was not significantly different between these two groups (Figure 5B). Total dermal damage and particularly adnexal damage was significantly higher in *Stephanofilaria* positive lesions (Figures 5C, D). Similarly, *Stephanofilaria*-infected lesions had significantly higher inflammation compared to non-infected lesions (Figure 5E). Although infiltration of eosinophils, neutrophils, macrophages, lymphocytes and plasma cells was evident in both lesion groups, the scores for eosinophils, macrophages and lymphocytes were significantly higher in lesions with *Stephanofilaria* (Figures 5F–J).

**Figure 6:**
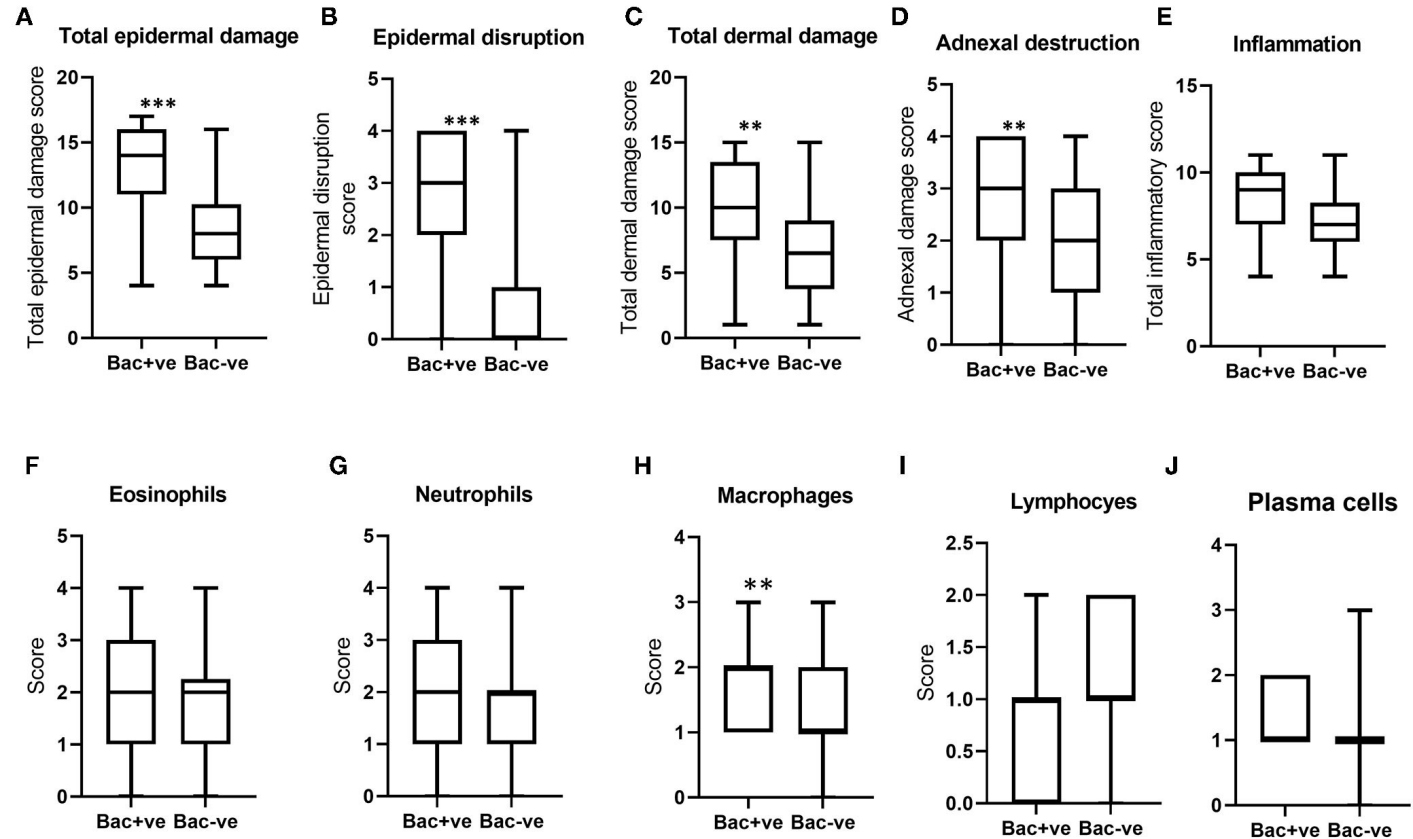
Boxplots **(A–D)** indicate the distribution of scores for total epidermal damage, epidermal disruption, total dermal damage and adnexal destruction for bacterial positive (Bac+ve) and negative (Bac-ve) lesions. Boxplots **(E–J)** show total inflammation and individual inflammatory cell scores of these two groups (**P < 0.01, ***P < 0.001).

When the histological scores for BF lesions with and without bacterial infection were compared, total epidermal damage, epidermal disruption, total dermal damage, and adnexal destruction in BF lesions with bacteria were significantly higher compared to non-infected lesions (Figures 6A–D). However, total inflammation scores and the differential leukocytic counts were not significantly different, except for macrophages, which were significantly higher in lesions where bacteria were present (Figures 6E–J). Notably, there was no difference in eosinophil count between lesions with and without bacteria, in contrast to the difference observed between *Stephanofilaria* positive and negative lesions.

**Figure 7:**
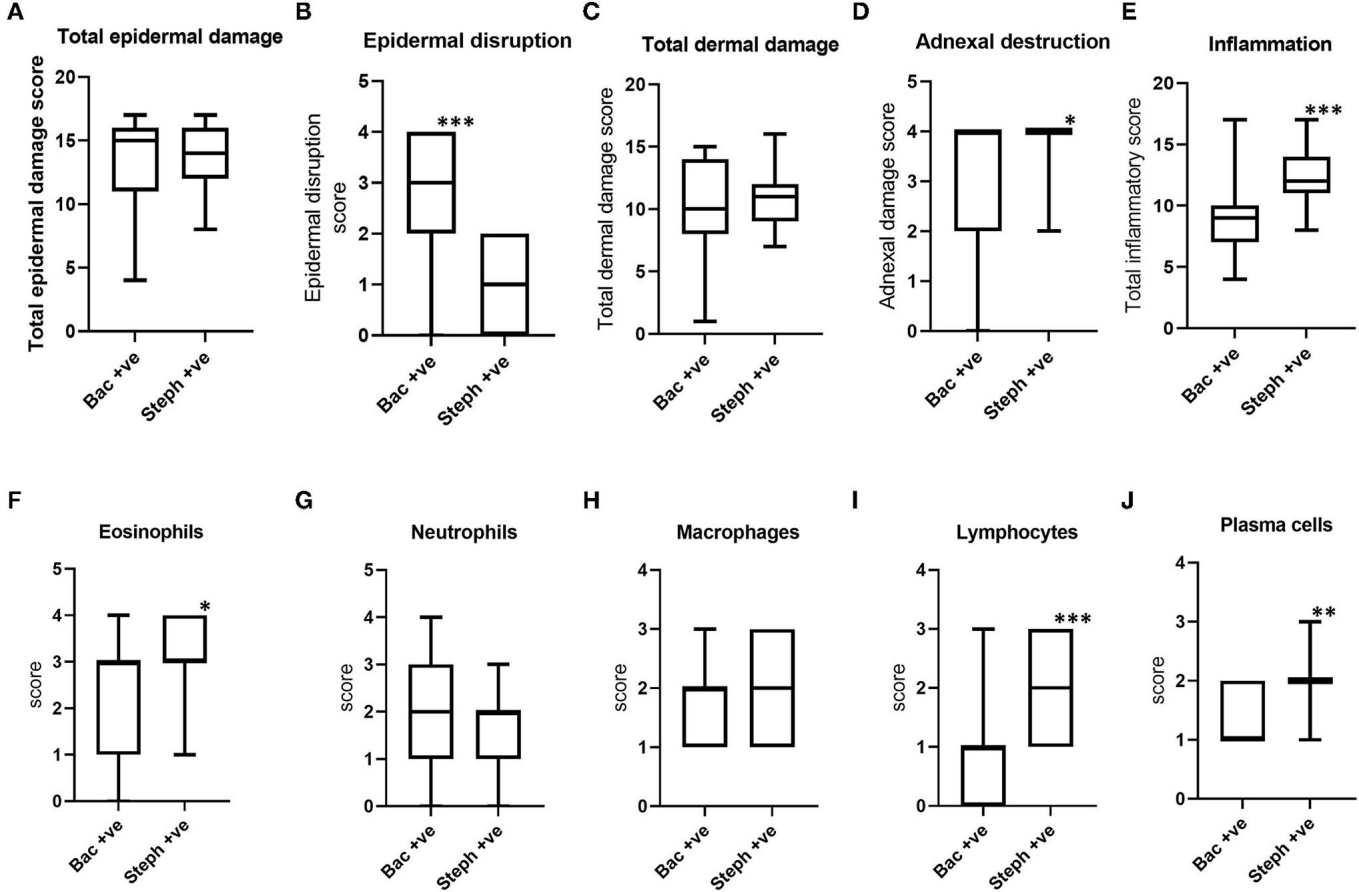
Boxplots **(A–D)** indicating the distribution of scores for total epidermal damage, epidermal disruption, total dermal damage and adnexal destruction scores for bacterial positive lesions (Bac+ve) and *Stephanofilaria* positive lesions (Steph +ve). Boxplots **(E–J)** show total inflammation and individual inflammatory cell scores of these two groups (*P < 0.05, **P < 0.01, ***P < 0.001).

When the histopathological scores of *Stephanofilaria* positive BF lesions were compared with bacteria-infected lesions, although epidermal disruption was significantly higher in lesions with bacteria (Figure 7B), total epidermal damage was not significantly different (Figure 7A). Out of 17 bacteria-positive lesions, only two had zero scores for epidermal disruption. The score for total dermal damage was not significantly different between bacteria and *Stephanofilaria* positive lesions (Figure 7C). Adnexal damage was significantly higher in *Stephanofilaria*-infected lesions than in bacteria-infected lesions (Figure 7D) and *Stephanofilaria*-infected lesions had a significantly higher inflammation score (Figure 7E). Infiltration of eosinophils, neutrophils, macrophages, lymphocytes, and plasma cells was evident in both lesion groups but the scores for eosinophils, lymphocytes and plasma cells were significantly higher in *Stephanofilaria*-infected lesions (Figures 7F–J).

## 4. Discussion

Our study has highlighted the complex interplay amongst BFs, *Stephanofiliaria* and bacteria in BF lesion development. Johnson et al. (4) were the first to report the association between BF lesions and *Stephanofilaria* infection, which at that time was considered to be the main etiological agent of these lesions. However, Johnson et al. (5) only detected *Stephanofilaria* in 40% of the lesions they examined, an observation which the authors (5) attributed to the low sensitivity of histology and saline extraction techniques they used, the only detection methods available at that time. It is notable however that in controlled studies Johnson (3) was able to induce lesions similar in appearance to field BF lesions by exposing cattle held in fly proof cages to high numbers of BF not known to be infected with *Stephanofilaria* sp.

The recent development of a more sensitive qPCR for detecting the presence of *Stephanofilaria* in lesions and BFs (18) provided further evidence that *Stephanofilaria* is not present in all BF lesions and indeed appears to be completely absent from BF populations in some regions where BF lesions are prevalent (10). The availability of this test provided the opportunity to further examine the importance of potential causal factors in the development of BF lesions. Our study extended on previous work, comprehensively describing and differentiating the gross and microscopic pathological features of BF lesions involving potential comorbid etiological factors including *Stephanofilaria* sp., bacteria, and host immune response.

When the size of the BF lesions which had *Stephanofilaria* was compared to bacterial infected lesions or lesions negative for both *Stephanofilaria* and bacteria, the area of the lesions with *Stephanofilaria* was significantly greater than for either of the other two groups, extending up to 54 cm^2^ area in some instances. However, even in the absence of both *Stephanofilaria* and bacteria, BF lesions could attain a lesion size up to 40 cm^2^ and it is hypothesized that this could be a function of individual animal hypersensitivity to BF antigens as has been previously suggested for HF-associated lesions in some instances (16, 20). In addition, the presence of BF lesions causes mild to severe pruritus (7) manifesting as frequent scratching and rubbing of the lesions which could also act to increase the lesion area and the severity of the tissue damage.

Hyperkeratosis and alopecia were more obvious gross features in *Stephanofilaria* infected lesions than in lesions without nematodes and there was no difference between bacteria-infected and non-infected lesions for these gross changes. These observations are consistent with previous reports for *Stephanofilaria*-infected lesions (21, 22) and consistent with our casual observations that hyperkeratotic hairless lesions are commonly present in cattle in northern Queensland where *Stephanofilaria* is prevalent, but not commonly seen in southern Queensland where no *Stephanofilaria* was found (10). The occurrence of ulceration in bacteria-infected lesions is consistent with observations of Devriese and Derycke (23) and Hazarika et al. (24), who isolated *Staphylococcus hyicus* from ulcerative cattle skin lesions. Although the difference in ulceration scores between bacteria positive and negative lesions was not significant in our study, ulceration was significantly higher in lesions with bacteria than in lesions with *Stephanofilaria*.

Epidermal disruption was the most noticeable epidermal change associated with the bacterial infection in our study, but was not evident in *Stephanofilaria*-infected lesions. Johnson (3) also observed breached epidermis associated with bacterial growth in some lesions, but in common with the current study rarely observed this change in the *Stephanofilaria*-infected lesions. Naseem et al. (25) isolated *Staphylococcus hyicus* and *S. agnetis* from BF lesions and both these bacterial species were found to have exfoliative toxin type A and C genes. These toxins are epidermolytic serine proteases that can digest skin desmoglein, destroying keratinocyte adhesion and can cause epidermal damage (26). Similar toxins have been identified in *S. hyicus* isolates associated with exudative epidermitis in pigs (“greasy pig disease”) (27). However, clinical expression of exfoliative toxins has not yet been confirmed and it is possible that these toxins are not actively involved in lesion development as we observed some uncircumscribed ulcerated lesions. Based on the isolation of *Staphylococcus* spp. from ulcerative BF lesions by Naseem et al. (25), it appears that the cocci-shaped bacterial colonies observed in this study are likely to be *S. hyicus* or *S. agnetis*, although *S. aureus* was also reported from similar lesions in dairy cattle associated with HF (17).

The varying degrees of hyperkeratosis and serocellular crust formation observed in all *Stephanofilaria* positive lesions are consistent with the epidermal changes described with typical *Stephanofilaria* spp. associated lesions (3, 21, 22). However, there was also a significant difference between bacteria negative and positive lesions in the hyperkeratotic score. Mild to moderate epidermal changes including spongiosis, hyperkeratosis, acanthosis, epidermal disruption and formation of serocelluar crust, were also noted in sections examined from BF lesions negative for *Stephanofilaria* and bacteria. Similar epidermal changes have also been reported by Mosca et al. (20) who attributed these lesions to an allergic response to HF feeding. The failure to detect either *Stephanofilaria* or bacteria in these lesions and the resemblance of these epidermal changes to those seen in the HF-associated lesions suggests that these changes could be immunopathological effects resulting from an immune response to BF feeding.

Both *Stephanofilaria*-infected and bacteria*-*infected lesions had more severe dermal damage and adnexal destruction than lesions without infection. Johnson (3) suggests that in *Stephanofilaria* infected lesions, this could be due to a severe localized host immune reaction, comprising histiocytes, lymphocytes and eosinophils, elicited by the adult nematodes present at the base of the hair follicle. A similar response was seen in our study where eosinophils were seen clustered around *Stephanofilaria* adult nematodes and microfilariae. This could subsequently result in the destruction of the hair follicles. Our study also observed complete loss or early signs of adnexal destruction in the BF lesions without *Stephanofilaria* infection. Thus adnexal destruction could result from an allergic response triggered by BF feeding in addition to the response to *Stephanofilaria*. Further evidence for this is provided by Mosca et al. (20) and Guglielmone et al. (28) who reported severe dermal oedema, folliculitis and furunculosis in HF-associated skin lesions without the presence of *Stephanofilaria*. There was also a significant difference in dermal damage and adnexal destruction scores between bacteria-infected and noninfected lesions, which suggest that bacterial infection could also play a role.

Although eosinophil infiltration in the superficial dermal layer was observed in all the BF lesions examined in this study, *Stephanofilaria* infection produced significantly higher eosinophilic infiltration, especially around the adult nematode and microfilariae. Eosinophilic dermatitis has also been reported by Whittier et al. (22) and Watrelot-Virieux and Pin (21) in *Stephanofilaria*-infected lesions. In contrast to our finding of significantly higher numbers of eosinophils, macrophages, and lymphocytes in *Stephanofilaria* positive lesions, Johnson (3) indicated eosinophils and neutrophils as the major inflammatory cells in lesions without *Stephanofilaria* whereas he observed histiocytes and lymphocytes were the predominant inflammatory cells in *Stephanofilaria*-infected lesions. The difference between our observations and those of Johnson (3) could be explained by difference in lesion stage when biopsied, as Patnaik (29) also observed lymphocytes and histiocytes dominated inflammatory response around dead worms in chronic infections of *Stephanofilaria assamensis*. The eosinophil-dominant inflammatory reaction in the BF lesions, without nematode or bacterial infection, may also indicate that hypersensitivity responses to BF feeding play a major role in lesion pathology, as a similar inflammatory pattern was reported for *Stephanofilaria* sp.-negative HF associated lesions (20, 28).

Overall our findings suggest that both *Stephanofilaria* and bacteria can play a role in BF lesion development. However, it appears that neither of these factors is essential for lesion development as we did not find either in 49% of the samples studied. This suggests that either there is a further unidentified factor involved, or that BF feeding can initiate lesion development without the involvement of other factors. *Stephanofilaria* infection caused more severe damage to the dermal layer, and could be a key factor in the formation of dry crusted type lesions often described in association with *Stephanofilaria* infection, whereas bacteria-infected lesions had more severe epidermal damage, which may drive the development of more open ulcerative lesions. Hypersensitivity to BF feeding is hypothesized to be an important contributing factor, particularly in the case of highly allergic individuals, as tissue damage and eosinophilic inflammation were important histological features in the absence of *Stephanofilaria* or bacteria. In addition, pruritis and rubbing of lesion areas, likely mediated by IgE based responses, may exacerbate the severity of lesions and resultant hemorrhage. Another possible explanation for the causality of lesions with unidentified co-factors could be the physical damage to skin caused by abrading mouthparts of BFs during feeding. As BFs have been shown to vector both *Stephanofilaria* (3, 8) nematodes and *Staphylococcus* spp. bacteria (25) they could also contribute to the development and severity of lesions by this means. Furthermore, lesions are often seen to persist well after the BF season has ended, suggesting that infection with *Stephanofilaria* or bacteria or any unknown factor may increase the longevity of lesions.

Treatment of cutaneous lesions associated with BF or HF, most commonly targeting *Stephanofilaria* nematodes, have given variable results (3, 14). Notably, the best effect has generally been seen with macrocyclic lactones that affect both the nematodes and flies (3, 14, 30), reported from the areas where both flies and *Stephanofilaria* are prevalent. Our results suggest that approaches that directly target BF, in addition to *Stephanofilaria*, could give more consistent treatment results and including a bacteriaocide, may also help to limit the severity of lesions.

## Data availability statement

The original contributions presented in the study are included in the article/supplementary material, further inquiries can be directed to the corresponding author.

## Ethics statement

The animal study was reviewed and approved by the University of Queensland Animal Ethics approval no. 2021/AE000054.

## Author contributions

MN, RA, and PJ contributed to conceptualization and design of study. MN, RA, AR, CC, MM, and MK contributed to methodology. MN and RA contributed to investigations and data visualization. MN and PJ contributed to statistical analysis. PJ, RA, AR, CT, MM, CC, and AT supervised this study. PJ and AT contributed to project administration and acquisition of funding. MN contributed to writing original draft, review, and editing. RA, CC, PJ, AR, AT, CT, and MM contributed to review and editing. All authors contributed to the article have approved the submitted version.
